# Cyclophilin A and C are the Main Components of Extracellular Vesicles in Response to Hyperglycemia in BV2 Microglial Cells

**DOI:** 10.1007/s12035-025-04921-6

**Published:** 2025-04-08

**Authors:** Noelia Castedo, Amparo Alfonso, Rebeca Alvariño, Mercedes R. Vieytes, Luis M. Botana

**Affiliations:** 1 Departamento de Farmacología, Facultad de Veterinaria, IDIS, Universidad de Santiago de Compostela, Lugo, 27002 España; 2 Departamento de Fisiología, Facultad de Veterinaria, IDIS, Universidad de Santiago de Compostela, Lugo, 27002 España

**Keywords:** Cyclophilins, Extracellular vesicles, Hyperglycemia, Microglia, Neuroinflammation

## Abstract

Cyclophilins (Cyps) and CD147 receptor play a crucial role in the inflammatory responses. Chronic inflammation causes tissue damage and is a common condition of several inflammation-based pathologies as diabetes or Alzheimer´s disease. Under high glucose (HG) conditions, microglia is activated and releases inflammatory mediators. In this process the role of Cyps is unknown, so this study was aimed to investigate the profile of Cyps in microglia and their release through extracellular vesicles (EVs) under hyperglycemia. An increase in reactive oxygen species (ROS) and nitric oxide (NO) levels was observed when BV2 glia cells were incubated with HG concentration. These effects were mitigated by the Cyps inhibitor cyclosporine A (CsA), suggesting the implication of Cyps in BV2 activation. In these conditions the intracellular expression of CypA, B, C and D, as well as the membrane expression of CD147 receptor was increased. In addition, only CypA and CypC were detected in the extracellular medium. Then, the presence of Cyps inside EVs was explored as an alternative secretion route. Interestingly, under HG treatment, an increase in the levels of the four Cyps in EVs was observed. When neurons were treated with EVs derived from HG-treated glia cells, their viability was reduced and EVs were detected in cytosol neurons pointing to an EVs-Cyps neurotoxic effect. These findings provide novel insights into the relationship between Cyps and EVs in neuroinflammation in hyperglycemia conditions. The current results strengthen the role of Cyps in cell communication and its potential role in brain function under pathological conditions.

**Supplementary Information:**

The online version contains supplementary material available at 10.1007/s12035-025-04921-6.

## Background

Glucose is the principal source of energy for body cells, specially brain cells [1]. The persistence of HG levels, or hyperglycemia, is the main hallmark of diabetes mellitus, a risk factor for both cardiovascular and Alzheimer’s diseases [2, 3]. Prolonged HG leads to an increased release of proinflammatory mediators and ROS, mainly produced during the mitochondrial respiration. Under physiological conditions, the antioxidant systems counteract ROS production but under HG, there is ROS overproduction that can cause damage to DNA, proteins, and other molecules. In addition, ROS generation leads to vascular inflammation, leukocyte adhesion and insulin resistance [4]. HG causes long-term complications and induces a response of the central nervous system (CNS), known as neuroinflammation, with inflammatory brain cells being activated and neurons starting to die [1, 5]. The key driver of this inflammatory response is microglia, which are resident macrophage-like immune cells in the CNS that represent the first line of host defense against brain damage [6]. These cells exhibit a wide phenotypic plasticity, ranging from a responding to stimuli phenotype to a surveillant phenotype [7]. Under physiological conditions, microglia is in a homeostatic state that changes to a state reactive to stimuli when immunological homeostasis is disrupted [6]. In this phenotype, the active microglia secretes proinflammatory mediators that increase neuronal death and compromise the blood–brain barrier (BBB) integrity, allowing peripheral inflammatory cells to infiltrate and increase the damage [6, 8]. HG induces the responding phenotype, affects the activity and the inflammatory status of microglia, and upregulates cytokines, chemokines, and adhesion molecules levels [8, 9]. In response to some proinflammatory and oxidative stimuli, microglial cells could also secrete other proteins such as Cyps [10, 11].

Cyps are immunophilins with peptidyl-prolyl *cis–trans* isomerase (PPIase) activity involved in protein folding and assembly, cell signaling and trafficking [12]. They participate in inflammatory processes but their main role in inflammation remains undescribed. There are more than 18 isoforms, all with affinity for CsA, an immunosuppressant drug produced by the fungus *Tolyplocadium inflatum* [13, 14]. Among all of them, this work is focused on CypA, CypB, CypC and CypD. CypA is the most abundant and is mainly located in the cytosol and nucleus of cells. CypA is accumulated in pericytes and brain endothelial cells from *APOE* carriers, which imply a higher risk of developing Alzheimer’s disease [15]. Likewise, CypA expression is increased in microglial cells in response to inflammatory stimuli [10]. In the extracellular medium, CypA acts as a chemokine, promoting the activation of proinflammatory cells, and as a paracrine and autocrine factor, mediating cell-to-cell communication through the CD147 receptor [16]. CD147 is a type I transmembrane glycoprotein belonging to the immunoglobulin superfamily which is upregulated in neuronal, glial, and endothelial cells under inflammatory conditions [10, 17]. CypA is also present in serum from patients suffering from metabolic and cardiac diseases, in which HG is one of the main hallmarks [18–21]. CypB is located in the cytosol, nucleus and endoplasmic reticulum (ER) of cells and is involved in redox homeostasis. CypB is released to the extracellular space by lymphocytes, where it interacts with CD147 and acts as a chemotactic agent of inflammatory cells into damaged tissues [17]. Nevertheless, CypB role is controversial since a protective effect against ROS and proinflammatory stimuli has been described [16]. CypB is increased in the serum from patients with hypertension, obesity and diabetes [22–24]. CypC is located in the Golgi apparatus, ER and in the extracellular space. It has a restricted tissue distribution compared to CypA and CypB and is involved in redox homeostasis [16, 25]. It is secreted to the extracellular space by T lymphocytes and adipocytes, playing a role in endothelial dysfunction [13, 17]. CypC interaction with CD147 is undescribed although an increase on the receptor expression after stimulation with CypC has been reported [26]. CypC was also defined as a possible marker for cardiovascular diseases, so this protein could play an important role in disorders with chronic inflammation [27]. CypD is the only Cyp that modulates the mitochondrial transition permeability pore opening, a channel that causes a massive outflow of ions, mitochondria swelling and cell death [28]. In the extracellular medium the role of CypD is poorly understood, but intracellularly it regulates the mitochondria bioenergetics and stabilizes the transition permeability pore opening during ischemia–reperfusion injury [16, 29, 30]. Furthermore, CypD deficient macrophages showed a decreased inflammatory response and the modulation of CypD in microglial cells reduced their activation [31, 32].

In addition to their presence in the extracellular medium, CypA and CypD were also detected in vesicles [33–37]. EVs are membranous structures with the same topology as the cell whose diameter varies between 30–200 nm. They are mainly produced via the endosomal pathway and released into the extracellular space by generating multivesicular bodies. EVs contain DNA, RNA, proteins, and other bioactive molecules and play critical roles in intracellular communication [38]. Several transmembrane proteins, called tetraspanins, such as CD63, have been used as markers of EVs due to their accumulation and can function as surface signaling molecules and mediate the inclusion of other proteins into EVs [38, 39]. EVs are released under both physiological or pathological conditions, transferring different proteins during cell-to-cell communication and in many inflammatory diseases. The content of EVs differ depending on the inflammatory stimuli and may have different functions [40].

In this context, the present study aimed to characterize the relationship between Cyps, CD147 receptor and HG in microglia cells. The intracellular levels of these proteins, as well as their release as free forms or inside EVs under hyperglycemia conditions will be explored. In this way, the role of Cyps in glia cells under inflammatory conditions will clarify their potential function in cellular communication and as biomarkers of pathologies associated with high levels of glucose and inflammation-based diseases.

## Materials and Methods

### Chemicals and Solutions

D (+)-Glucose anhydrous (C_6_H_12_O_6_) was purchased from PanReac AppliChem (Barcelona, España). Polyacrylamide gels and molecular weight marker Precision Plus Protein Standards Kaleidoscope were obtained from Bio-Rad (Barcelona, Spain). Protease Inhibitor Complete Tablets and Phosphatase Inhibitor Cocktail Tablets, Total Exosome Isolation Reagent, Exosome-Depleted Fetal Bovine Serum, Griess Reagent Kit, carboxy-H_2_DCFDA, BODIPY TR Ceramide, SYTO RNASelect Green dyes and cells culture mediums were purchased from Thermo Fisher Scientific (Madrid, Spain). ELISA kits were purchased from Abbexa (Cambridge, UK) and MyBioSource (San Diego, USA) and CsA with a purity ≥ 98.5% is from Abcam (Cambridge, UK). Polyvinylidene difluoride (PVDF) membrane, bovine serum albumin, Lipopolysaccharide (LPS) from *Escherichia coli* O111:B4 and the rest of chemicals and reagents were obtained from Sigma-Aldrich (Madrid, Spain). The composition of the saline solution (Locke’s Buffer) used for the viability assays was (in mM): 154 NaCl, 5.6 KCl, 1.3 CaCl_2_, 1 MgCl_2_, 3.6 NaHCO_3_, 5.6 Glucose and 10 HEPES. Griess reagent used to determine the NO levels contained 1% sulfanilamide in phosphoric acid (5%) and 0.1% naphtylethylenediamine dihydrochloride. The composition of the EVs lysis buffer (RIPA buffer) was: 150 mM NaCl, 1% Triton X- 100, 0.5% sodium deoxycholate, 0.1% sodium dodecyl sulphate (SDS), 50 mM Tris–HCl (pH 8.0) and phosphatase/protease inhibitors cocktails. The composition of the buffer used to lysis the cytosolic fraction was (in mM): 20 Tris–HCl (pH 7.4), 10 NaCl and 3 MgCl_2_, containing phosphatase/protease inhibitors cocktails. The phosphate buffered saline (PBS) solution was composed by: 137 mM NaCl, 8.2 mM Na_2_HPO_4_, 1.5 mM KH_2_PO_4_ and 3.2 mM KCl.

### Cell Culture

Murine microglial BV2 cell line was purchased from Interlab Cell Line Collection (ICLC), number ATL03001. Cells were maintained in RPMI 1640 medium with L-Glutamine and phenol red supplemented with 10% fetal bovine serum (FBS), penicillin (100 U/mL), and streptomycin (100 μg/mL). Cells were dissociated twice a week using 0.05% trypsin/EDTA.

Neuroblastoma SH-SY5Y cell line was purchased from American Type Culture Collection (ATCC), number CRL2266. Cells were maintained in Dulbecco’s modified Eagle Medium: Nutrient Mix F- 12 (DMEM/F- 12) supplemented with 10% FBS, 1% glutamax, penicillin (100 U/mL), and streptomycin (100 μg/mL). Cells were dissociated weekly using 0.05% trypsin/EDTA.

Both cell lines were maintained at 37 °C in a humidified atmosphere of 5% CO_2_ and 95% air.

### Cell Viability Assay

Cells were seeded in 96-well plates at a density of 4 × 10^4^ cells per well for 24 h. Cells were treated with different HG concentrations (25–50 mM) for 24 h. Cells were pretreated 1 h with CsA (1 μM), if applicable. Effect of these treatments on the viability was determined via 3-(4,5-dimethylthiazol- 2-yl)− 2,5-diphenyl tetrazolium bromide (MTT) assay [11]. After treatment, cells were washed three times with saline solution and 200 μL of MTT (500 μg/mL) dissolved in saline buffer were added to each well. After 1 h of incubation at 37 °C, cells were disaggregated with 5% SDS. Formation of formazan crystals was measured at 595 nm with a spectrophotometer plate reader. Saponin at 1 mg/mL was used as death control. Experiments were performed by triplicate at least three independent times.

### Measurement of Reactive Oxygen Species Levels

Intracellular ROS levels were determined with carboxy-H_2_DCFDA dye, as previously described [31]. A total of 4 × 10^4^ microglial cells per well were seeded in 96-well plates for 24 h at 37 °C. Cells were treated as described before for the viability assay. After 24 h-treatment, cells were washed twice with RPMI medium without FBS before adding 200 μL of 20 μM carboxy-H_2_DCFDA. After 1 h of incubation at 37 °C, the dye was removed and 200 μL of PBS were added. Cells were incubated for 30 min at 37 °C, and ROS release was measured at 495 nm excitation and 527 nm emission with a spectrophotometer plate reader. All measurements were performed in triplicate at least three independent times.

### Determination of Nitric Oxide Levels

NO levels were analyzed with Griess method, which detects the formation of nitrite by the spontaneous oxidation of NO [41]. Cells were cultured in 12-well plates at a density of 1 × 10^6^ cells per well in cell culture medium without phenol red for 24 h at 37 °C. Microglial cells were treated with HG (25 mM) for 24 h and pretreated 1 h with 1 μM CsA, if necessary. Then, 150 μL of culture medium were mixed with 50 μL of Griess reagent. After a 30 min-incubation at room temperature and darkness, nitrite formation was determined by measuring absorbance at 546 nm with a spectrophotometer plate reader. All measurements were performed in triplicate at least three independent times.

### Cytokine Secretion from BV2 Cells

IL- 6 release by BV2 cells was determined by measuring the cytokine levels in the cell culture supernatant with an ELISA kit following the manufacturer’s instructions. Briefly, BV2 cells were seeded in 96-well plates at a density of 4 × 10^4^ cells per well for 24 h. Microglia cells were pretreated 1 h with 1 μM CsA, if applicable, and then treated with HG (25 mM) for 24 h. LPS (500 ng/mL) was used as positive control of IL- 6 release. All samples were run in duplicate and the concentration was calculated using a standard curve. To obtain the optical density of each well, a spectrophotometer microplate reader was used at a wavelength of 450 nm.

### CD147 Receptor Expression in BV2 Cells Surface

BV2 microglial cells were seeded at a density of 1 × 10^6^ cells per well in 12-well plates. After 24 h of HG treatment, CD147 receptor expression was assessed by flow cytometry analysis. Briefly, cells were washed twice with 500 μL PBS-BSA (5%). Then cells were incubated with 5 μL of Anti-Mouse CD147 conjugated with FITC (ThermoFisher, Madrid, Spain) for 60 min in darkness at 4 °C and 150 rpm. After that, cells were washed and resuspended in 100 μL PBS with 1% of paraformaldehyde. A total of 10,000 events were measured at wavelengths of 498 nm excitation and 517 nm emission by the Amnis ImageStream X MKII (Luminex corporation) multispectral imaging flow cytometer. To analyze and represent the data obtained, the IDEAS ImageStream Analysis software v6.0 was used. All measurements were performed by duplicate three independent times.

### Extracellular Vesicles Isolation

BV2 cells were seeded at a density of 2 × 10^6^ cells per well in 6-well plates and treated for 24 h as described above. After treatment, the supernatant of each well was centrifuged for 30 min at 4 °C and 3100 rpm. Supernatant was filtered through 0.22 μM filters and then concentrated with ultracentrifuge tubes (Amicon Ultra Centrifugal Filter, 30 kDa MWCO from Sigma-Aldrich). The concentrated was transferred to eppendorfs and resuspended with the Total Exosome Isolation Reagent (500 μL per milliliter of supernatant). After overnight incubation at 4 °C, tubes were centrifuged for 1 h at 4 °C and 13,000 rpm. The supernatant was discarded and the pellet containing EVs was resuspended in 60 μL PBS and stored at − 20 °C. In order to analyze the protein content, 60 μL of RIPA buffer containing phosphatase/protease inhibitors were added to isolated EVs. Samples were centrifugated for 10 min at 4 °C and 6000 rpm and protein concentration was determined by Bradford method. Experiments were performed three independent times.

### Extracellular Vesicles Characterization

Nanoparticle tracking analysis (NTA) was used to characterize the size and concentration of EVs from BV2 microglial cells by measuring the movement of EVs through image analysis. Briefly, 1 mL of EVs pellet-PBS resuspended in RNase-free water (1:100 dilution factor) was injected into the NanoSight NS300 instrument (Malvern, UK) equipped with a 488 nm blue laser and sCMOS camera. NanoSight software (NTA 3.4 Build 3.4.003) was used to analyze the samples and record five 60-s videos using the following settings: syringe pump speed 40, camera level 14, temperature 25 °C and viscosity 0.9 cP (water). The analysis settings were as follows: the detection threshold was set to 17 and the focus was manually adjusted.

Purified EVs were fixed with 2% paraformaldehyde to visualize them through Transmission Electronic Microscopy (TEM). Briefly, 1 mL of 2% paraformaldehyde was added to the EVs pellet for 5 min. Then, samples were centrifuged at 4 °C and 13,000 rpm for 1 h. Supernatant was discarded and 100 μL PBS were added before preparing the samples for TEM.

### Cytosolic Protein Extraction and Western Blotting

Cells were cultured at 1 × 10^6^ cells per well in 12-well plates and treated for 24 h as previously described. To obtain cytosolic protein fraction, cells were washed twice with ice-cold PBS and 100 μL of lysis buffer were added to each well. Then, cells were incubated on ice for 10 min and 5 μL of Triton X- 100 were added. After that, cells were centrifuged at 4 °C and 3000 rpm for 10 min. The supernatant obtained was collected as the cytosolic fraction and quantified through Direct Detect system (Merck, Germany).

For western blot experiments, 15 μg of cytosolic protein and EVs content or 15 μL of filtered extracellular medium were resolved on 4–20% polyacrylamide gels. Precision Plus Protein Standard Kaleidoscope molecular weight marker was used to determine the protein weight. Proteins were transferred onto PVDF membranes via Trans-Blot semi-dry transfer system. Membrane blockage with 0.5% BSA and antibody incubation were carried out using SNAP i.d. system (Merck). CypA was detected with anti-PPIA primary antibody (1:1000, Elabscience), CypB was quantified with anti-PPIB (1:1000, Elabscience), CypC was recognized with anti-PPIC (1:1000, Elabscience) and CypD was detected with anti-cyclophilin F primary antibody (1:1000, Abcam, UK). Cyps antibodies specificity was previously tested [42]. Specific protein bands were detected using the Supersignal West Pico or Supersignal West Femto substrate. Protein band intensity was normalized using anti-GAPDH (1:1000, Merck) in cytosolic lysates and anti-CD63 (1:1000, Abcam) in EVs lysates. Densitometry analysis of protein bands was performed using the Diversity GeneSnap software (Syngene, UK). All measurements were performed in duplicate three independent times.

### Cyps Levels Measurement in Culture Medium by ELISA

Extracellular Cyps levels were measured in culture medium from BV2 cells. Cyps measurements were done using mouse ELISA kits following the manufacturer’s instructions as previously described [43]. The concentrations were calculated using a standard curve, and all samples were performed in duplicate. To obtain the optical density of each well, a spectrophotometer microplate reader was used at a wavelength of 450 nm.

### Treatment of SH-SY5Y Cells with EVs Derived from BV2 Cells

Neuroblastoma SH-SY5Y cells were seeded at 5 × 10^4^ cells per well in 96-well plates and differentiated to neurons by retinoic acid (10 μM) treatment for 7 days [44]. New media was added every two days. Undifferentiated and differentiated cells were treated for 24 h with EVs isolated from BV2 cells as explained in the “Extracellular vesicles isolation” section.

### Extracellular Vesicles Staining and Uptake by Differentiated SH-SY5Y Cells

To stain the EVs, isolated vesicles derived from BV2 cells were incubated with 1 mM BODIPY TR Ceramide or SYTO RNASelect Green dyes for 20 min at 37 °C. Then, the volume was transferred to Exosome Spin Columns (MW 5,000) (Sartorius, Germany) and centrifuged at 15,000 rpm for 15 min at 4 °C to remove unincorporated dye. The labelling efficiency was analyzed in a fluorescence reader and it was above 90%. Then, 100 μL of labeled EVs were added to differentiated neuroblastoma cells for 3 or 24 h. Then, neurons were washed with PBS and fixed with 4% paraformaldehyde at room temperature for 10 min. Afterward, cells were incubated 20 min with PBS- 2% BSA- 0.1% Triton X100 and Hoescht for nucleus labeling (when SYTO RNASelect Green EVs were present), or Texas Red DNase I for G-actin labeling (when BODIPY TR Ceramide EVs were present).

Confocal images were acquired in a Nikon Eclipse TE2000-E inverted microscope attached to the C1 laser confocal system (EZC1 V.3.60 software; Nikon Instruments Europe B.V., Netherlands) with a 40X oil immersion objective. 405, 561 and 488 nm lasers were used for dyes excitation. Fluorescent images were acquired at a resolution of 512 × 512 pixels separately for each fluorophore and then mixed to avoid interferences. No interferences of Hoescht in red channel and BODIPY TR in blue channel were observed, as well as no interferences of Texas Red in green channel and SYTO RNASelect in red channel were detected.

### Statistical Analyses

Data are presented as mean ± SEM. Statistical differences were evaluated by one way ANOVA with Dunnett’s or Tuckey’s post hoc test using the GraphPad Prism v.10 software. Statistical significance was considered at *p* < 0.05.

## Results

Previous results indicate a possible role of Cyps in the inflammatory response underlying HG, since high levels of Cyps in serum from patients with cardiovascular diseases were associated with increased glucose levels [23]. Moreover, on the phase of the menstrual cycle where glucose concentration was higher, Cyps levels were also enhanced compared to the other phases [24]. Nevertheless, no data are available regarding CypA, CypB, CypC and CypD levels on brain cells under HG conditions. In this context, the aim of this work was to determine the relationship between Cyps and HG in BV2 microglial cells.

**Fig. 1:**
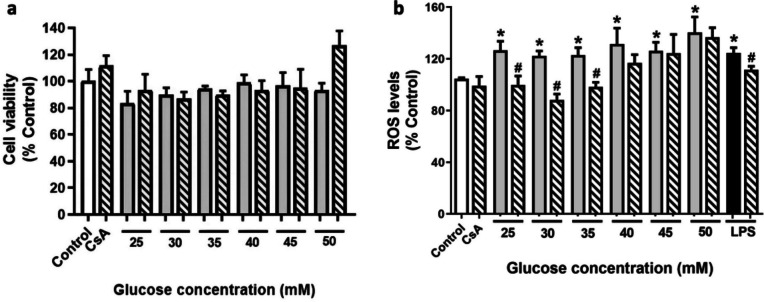
Effect of HG treatment in the activation of BV2 cells. Cells were stimulated with different HG concentrations (25–50 mM) for 24 h (grey bars) and pretreated with 1 μM CsA (striped bars) for 1 h in the same conditions. LPS at 500 ng/mL was used as positive control (black bar). **a** Cell viability of BV2 cells under HG conditions determined by MTT assay. **b** ROS release by microglia under HG. Results are mean ± SEM of three independent replicates performed by triplicate. Data are expressed as percentage of control cells, seeded in RMPI medium (11 mM glucose). Statistical differences determined by One way ANOVA’s test and Tuckey’s post hoc test. * *p* < 0.05 compared to control cells, # *p* < 0.05 between groups

First, the effect of HG treatment on cell viability was analyzed through MTT assay. Cells are usually cultured in RPMI medium with 11 mM glucose, therefore higher glucose concentrations were selected to study the impact of HG on microglia. Based on previous studies, cells were treated with different HG concentrations (25–50 mM) for 24 h [45]. Under the same conditions, cells were pretreated 1 h with 1 μM CsA, as previously described [11]. CsA is an immunosuppressant drug that targets Cyps, so the effect of CsA in combination with glucose on microglia viability was also analyzed. As observed in Fig. 1a, none of the treatments affected microglial viability compared to control cells (11 mM glucose).

BV2 cells are in a reactive state in HG conditions, as previously reported [45]. Microglial activation promotes oxidative stress and DNA damage, which leads to an overproduction of ROS and mitochondrial dysfunction together with neuron cell death and neurodegeneration. Therefore, the next step was to determine if microglial activation under these conditions was releasing ROS [6]. In that way, LPS was used as a control of inflammation since microglia secretes high amounts of ROS with this stimulus [46]. In addition, CsA was used as a positive control of Cyps inhibition. Results show that all the HG concentrations increased ROS (*p* < 0.05), reaching levels between 133% at 50 mM and 122% at 30 mM (Fig. 1b). At the lower HG concentrations (25, 30 and 35 mM), this effect was significantly decreased when cells were pre-treated with 1 µM CsA (*p* < 0.05). As expected, LPS increased ROS levels up to 124%, which were decreased after CsA pretreatment. In view of these results, 25 mM glucose (450 mg/dL) was the concentration chosen to perform the next experiments since it mimics a severely uncontrolled hyperglycemic state [47].

**Fig. 2:**
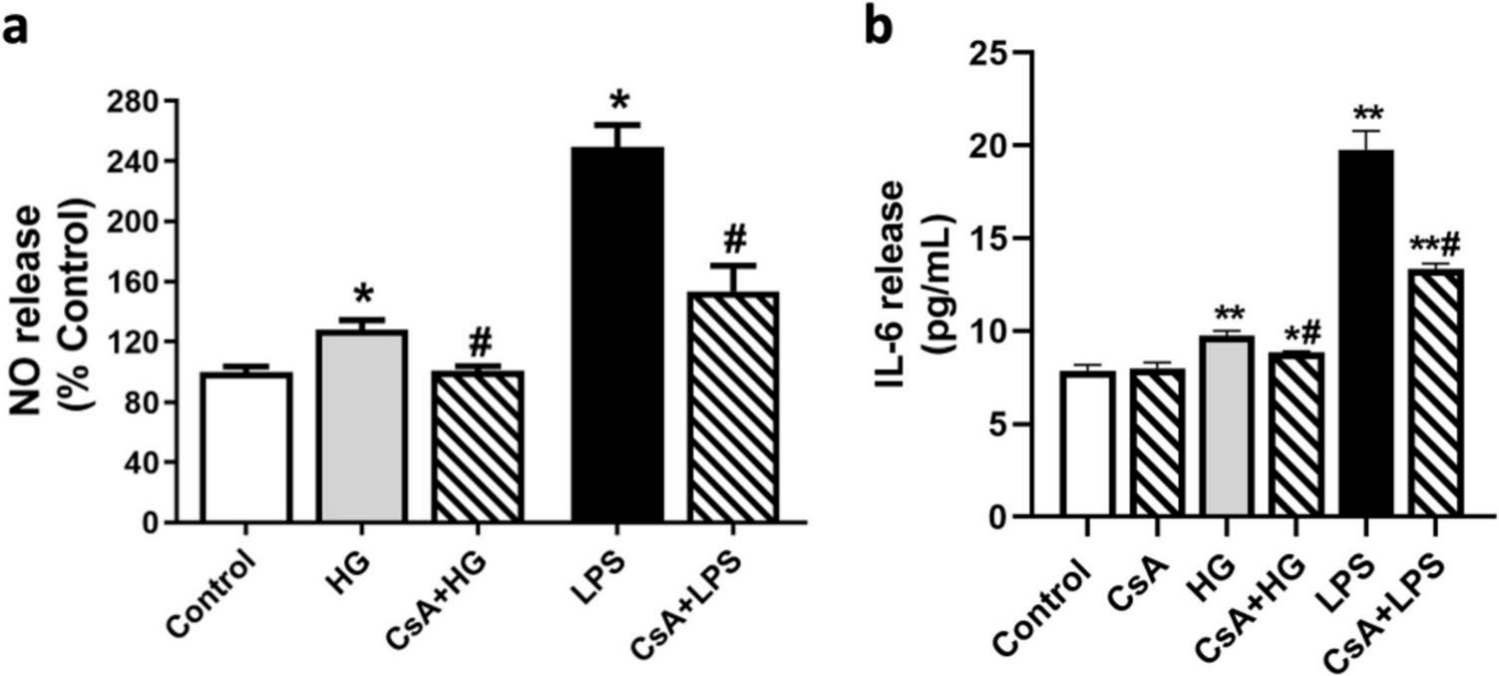
Effect of HG in the inflammatory response by BV2 cells. **a** NO release and (**b**) IL- 6 cytokine secretion by BV2 cells under HG conditions. Cells were stimulated with 25 mM glucose (HG) for 24 h and pretreated 1 h with CsA (1 μM), if applicable (striped bars). LPS (500 ng/mL) was used as positive control. NO release was assessed by Griess method and IL- 6 secretion by ELISA kit. Data are mean ± SEM of three independent replicates performed by triplicate. Results are expressed as percentage of control cells. Statistical differences determined by One way ANOVA’s test and Tuckey’s post hoc test. *p < 0.05, **p < 0.01 compared to control cells. # *p* < 0.05, ## *p* < 0.01 between groups

Under inflammatory conditions, BV2 cells are reactive to these stimuli and subsequently release high NO and IL- 6 levels, which exacerbates inflammation and oxidative stress contributing to neuron destruction [48, 49]. In this context, the following step was to analyze NO and IL- 6 release under HG conditions. Nitrite concentration was measured in the medium of BV2 cells after 24 h of treatment. As observed in Fig. 2a, BV2 cells released high amounts of NO to the cell medium under HG conditions (128 ± 6.2%), which were lower than under LPS treatment (249.4 ± 14.7%). Interestingly, NO secretion was significantly reduced when cells were pre-treated 1 h with 1 μM CsA in both HG (106.6 ± 6.2%, *p* < 0.05) and LPS conditions (173.4 ± 22.2%, *p* < 0.05). IL- 6 secretion to the extracellular medium was analyzed after 24 h of treatment (Fig. 2b). Under HG conditions, IL- 6 secretion was significantly increased (9.77 ± 0.24 pg/mL) compared to control cells (7.88 ± 0.33 pg/mL), an effect mitigated by CsA pretreatment (8.88 ± 0.06 pg/mL). Moreover, LPS stimulation also enhanced IL- 6 levels (19.78 ± 1.00 pg/mL), reduced when cells were pretreatment with 1 μM CsA (13.34 ± 0.30 pg/mL). These results together with ROS assay, show that BV2 response to HG conditions is mediated by Cyps, since their inhibition with CsA reduced ROS, NO and IL- 6 levels, as under LPS-associated inflammation.

**Fig. 3:**
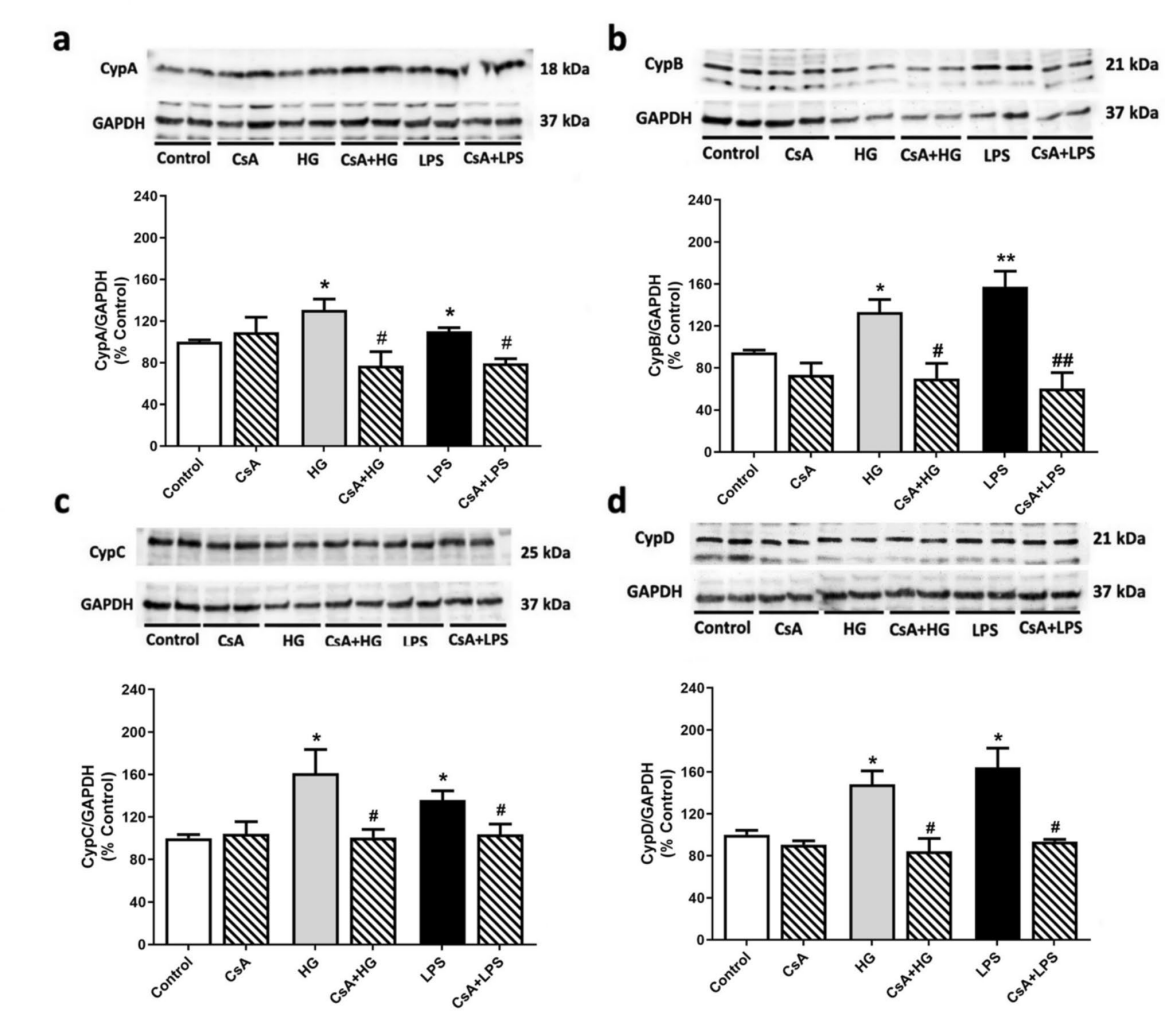
Intracellular expression under HG of (**a**) CypA, **b** CypB, **c** CypC and (**d**) CypD. Cells were stimulated with 25 mM glucose (HG) for 24 h (grey bars) and pretreated with 1 μM CsA for 1 h (striped bars), if applicable. LPS at 500 ng/mL (black bar) was used as positive control. Band intensity was normalized by GAPDH. Data are mean ± SEM of three independent replicates performed by duplicate. Data are expressed as percentage of control cells (11 mM glucose). Statistical differences determined by One way ANOVA test and Tuckey’s post hoc test. *p < 0.05, **p < 0.01 compared to control cells. # *p* < 0.05, ## *p* < 0.01 between groups

The next step was to confirm if Cyps were involved in HG response. In that way, intracellular Cyps expression under HG conditions was analyzed by western blot. When intracellular CypA expression was studied, a significant increase was detected under HG compared to control cells (130.7 ± 10.3%) while CsA treatment reduced CypA levels in these conditions by 53% (Fig. 3a). The intracellular expression of CypB was also enhanced in HG treatment compared to the control (126.7 ± 14.1%). In these conditions, CsA pretreatment decreased the microglial CypB expression by 44% (Fig. 3b). CypC intracellular expression on BV2 cells under HG was 60% higher than in control cells. This effect was 40% lower when microglia was pretreated with CsA (Fig. 3c). Intracellular CypD expression was also increased in microglia under HG (148.1 ± 12.9%) and reduced when microglia was pretreated 1 h with 1 μM CsA (84.4 ± 12.15%) (Fig. 3d). When cells were treated with an inflammatory stimulus (LPS), Cyps intracellular expression was increased compared to control cells. In the same conditions, 1 μM CsA pretreatment diminished the expression of the four proteins. Therefore, HG increases the intracellular expression of all four Cyps in BV2 microglial cells in a similar way than an inflammatory stimulus.

**Fig.4:**
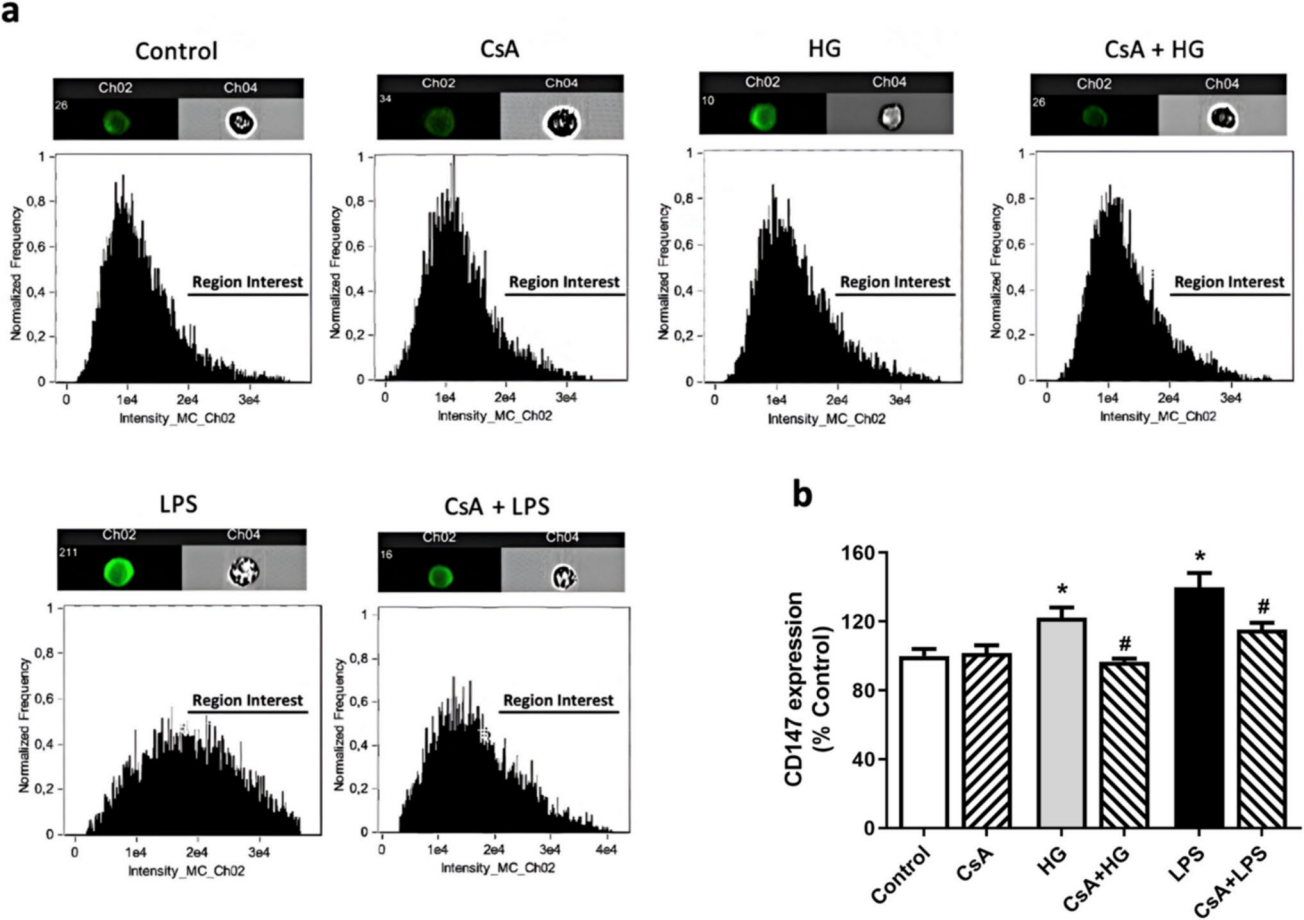
Effect of HG on CD147 receptor membrane levels in murine BV2 cells. Cells were treated with 25 mM glucose (HG) for 24 h and pretreated with 1 μM CsA in the same conditions. LPS at 500 ng/mL was used as positive control. **a** Representative images and histograms of CD147 receptor expression in surface membrane. **b** Graphic representation of results. Data are mean ± SEM of three independent replicates performed by duplicate. Results are expressed as percentage of control cells. Statistical differences determined by One way ANOVA test and Tuckey’s post hoc test. * *p* < 0.05 compared to control cells. # *p* < 0.05 between groups

CD147 is the primary signaling receptor for both extracellular CypA and CypB. This receptor is externalized on the cell surface when higher Cyps levels are detected [13]. Therefore, CD147 receptor expression in microglial membrane under HG conditions was analyzed. The results show that CD147 receptor was upregulated after 25 mM glucose treatment compared to control cells (122.3 ± 5.9%), as well as under LPS-induced inflammation (Fig. 4) [17]. In both conditions, CsA pretreatment reduced CD147 expression to control levels.

**Fig. 5:**
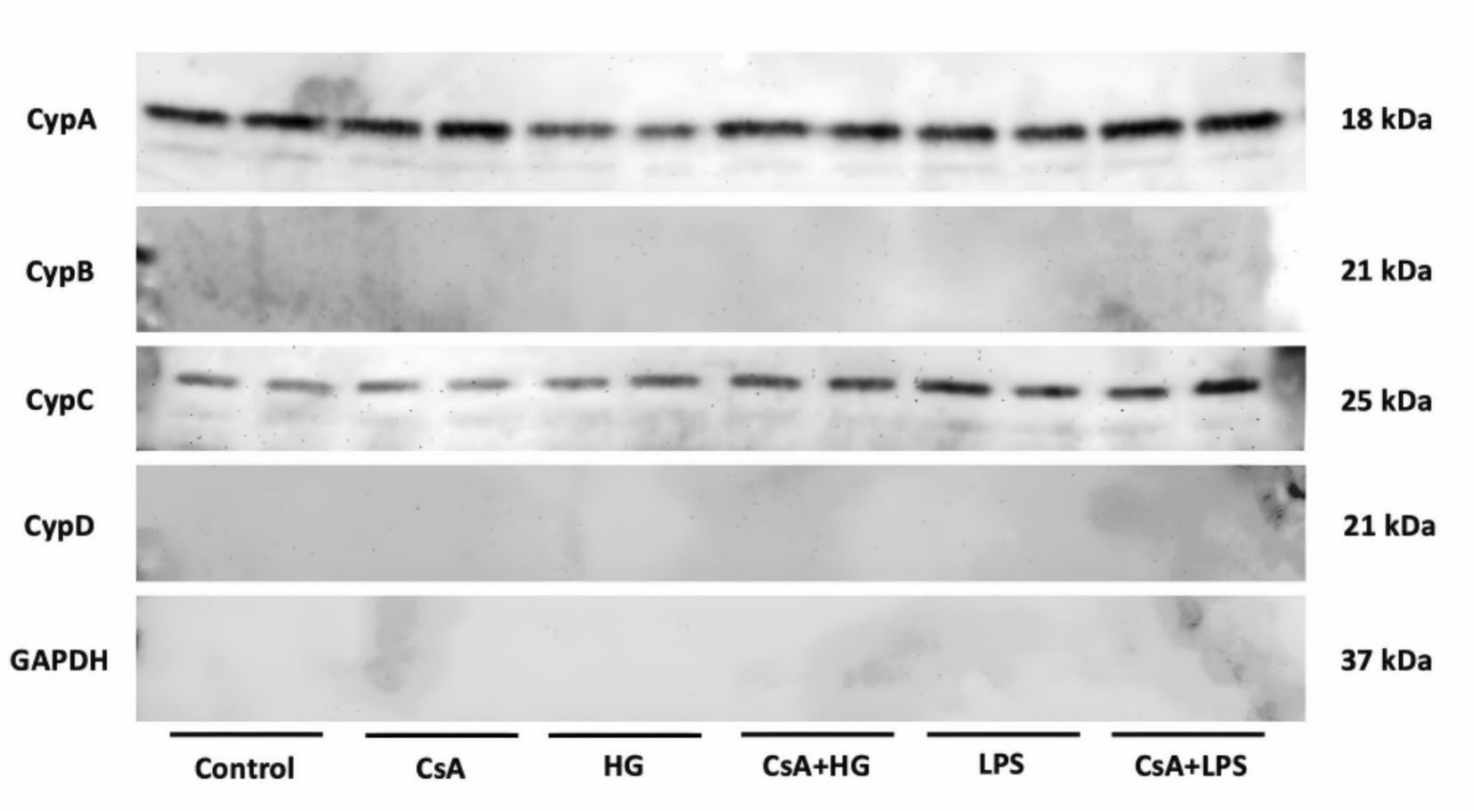
Cyps secretion to extracellular medium under inflammatory conditions. Cells were treated with HG for 24 h and pretreated 1 h with 1 μM CsA if applicable. LPS at 500 ng/mL was used as positive control. GAPDH was used as a negative control of cell supernatant samples

The next step was to evaluate Cyps release to the extracellular medium because these proteins participate in the communication between cells [16]. ELISA kits were conducted to measure Cyps levels in the medium from BV2 cells, but the proteins were undetectable. Consequently, the medium was filtered through 0.22 μM filters and concentrated with ultracentrifuge tubes (Amicon Ultra Centrifugal Filter, 30 kDa MWCO and 10 kDa MWCO) to increase protein concentration. Cyps levels were measured by ELISA in the concentrated medium but remained undetectable. Therefore, Cyps release to the extracellular medium under HG conditions was determined through western blot technique. Both CypA and CypC were detected in the medium under HG and LPS treatment. CypB and CypD were not detected in the medium of BV2 cells under any of the conditions, even after a stimulation with LPS (Fig. 5).

**Fig. 6:**
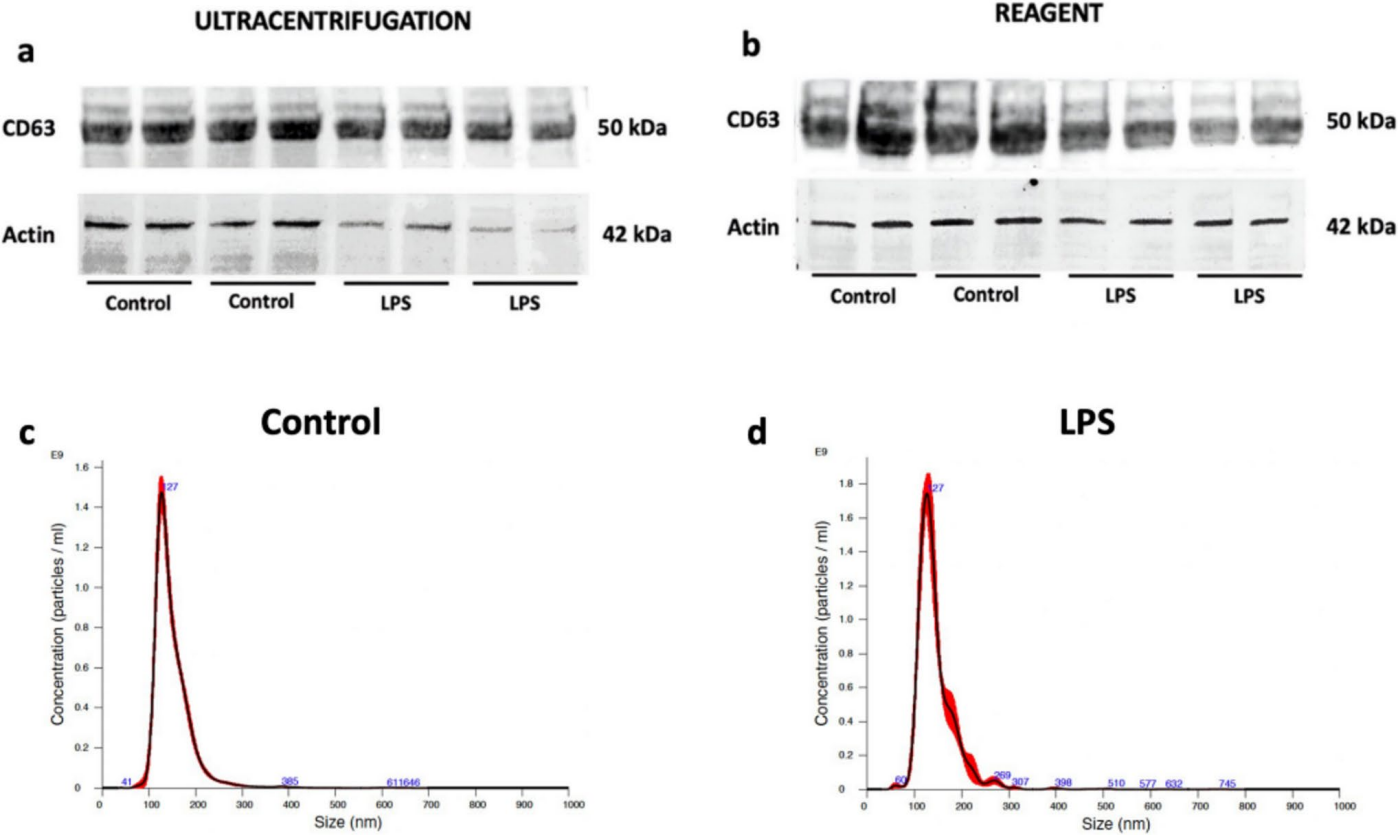
EVs release from BV2 microglial cells. Cells were treated with LPS (500 ng/mL) as control of inflammation. EVs presence was determined through the expression of CD63 and actin in both (**a**) ultracentrifugation and (**b**) reagent samples. **c** and **d** EVs characterization by NTA

As CypB and CypD were not detected in the medium, Cyps presence inside EVs was studied to analyze if they were released through these pathways. In this sense, CypA and CypD had been detected inside vesicles from other cell models under inflammatory conditions [36, 37]. First we determined if EVs were released from BV2 cells under inflammatory conditions [40]. Therefore, BV2 cells were treated 24 h with LPS and two methods, ultracentrifugation (100,000 rpm, 90 min, 4 °C) and a precipitation reagent (Total Exosome Isolation Reagent), were initially tested to precipitate and isolate vesicles following bibliographic references [50, 51]. According to the minimal requirements for detecting the presence of EVs, it is recommended to analyze the expression of at least one membrane marker in combination with EVs visualization by imaging techniques [52]. CD63 is a transmembrane protein, belonging to the family of tetraspanins, used as marker of EVs due to its accumulation [39]. So, CD63 expression in the EVs samples processed with both isolation techniques was analyzed by western blotting. CD63 was detected under control and inflammation conditions in all samples (Fig. 6a and b). Actin expression was also studied since it is an inclusive criterion to determine EVs purity [52], and as shown in Fig. 6 it was present on samples processed with both techniques. Following these criteria, the presence of albumin is an exclusion marker. Therefore, this protein was analyzed using the Ponceau Tinction. Since EVs isolated by ultracentrifugation contained albumin (MW: 65 kDa), the reagent was chosen to conduct the final assays (Supplementary Fig.S1). Finally, EVs from BV2 cells were characterized by NTA technique (Fig. 6c and d) and visualized by TEM (Supplementary Fig. S2). As Fig. 6c and d show, an homogeneous population of EVs is released by BV2 cells either in control conditions or after LPS treatment (mode 126.7 ± 1.0 nm and mode 126.3 ± 2.9 nm respectively).

**Fig. 7:**
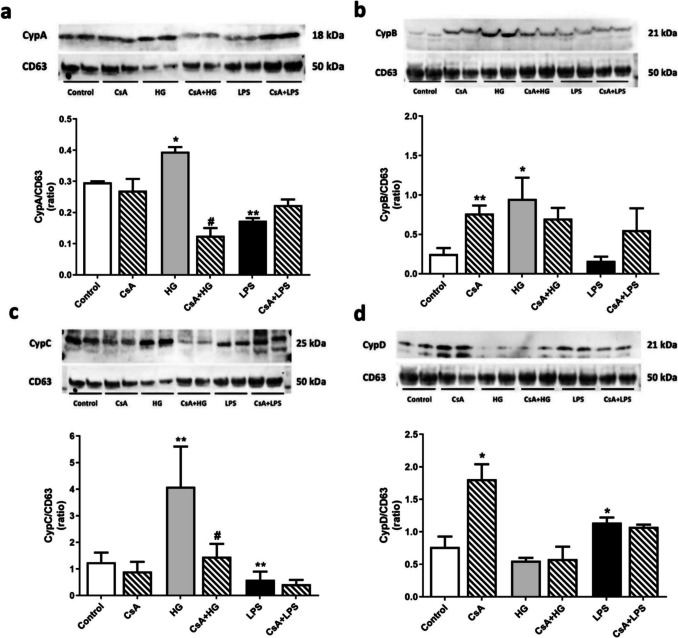
Cyps content inside EVs derived from microglia under HG conditions. **a** CypA, **b** CypB, **c** CypC and (**d**) CypD levels inside EVs. Cells were stimulated with 25 mM glucose (HG) for 24 h and pretreated (striped bars) with 1 μM CsA for 1 h if applicable. LPS (500 ng/mL) was used as positive control of EVs release (black bar) and CsA (striped bars) as internal control of Cyps route. Band intensity was normalized by CD63, a surface EVs protein. Data are mean ± SEM of three independent replicates. Data are expressed as ratio of Cyps content normalized by CD63 levels inside EVs. Statistical differences determined by One way ANOVA test and Tuckey’s post hoc test. *p < 0.05, ** *p* < 0.01 compared to control cells. # *p* < 0.05 between groups

The next step was to determine if HG also induced EVs release and if Cyps were present inside the vesicles. First EVs concentration was determined by NTA analysis. Interestingly HG and LPS treatments increase the number of EVs released (1.03 e + 11 ± 1.35e + 09 particles/mL and 1.03 e + 11 ± 1.40e + 09 particles/mL respectively) compared to control cells (8.30 e + 10 ± 2.18e + 09 particles/mL) (Supplementary Fig. S3). Then Cyps content inside EVs was checked. As Fig. 7a shows, CypA was significantly increased inside EVs in HG treatment compared to control cells (135 ± 9.4%). When cells were pretreated with 1 μM CsA, CypA content inside EVs was significantly reduced (42.7 ± 8.4%). CypB cargo inside EVs was also analyzed, and it was five times higher under HG conditions compared to control cells, as observed in Fig. 7b. CypB presence under HG treatment was reduced when cells were pretreated with CsA. As shown in Fig. 7c, CypC levels inside EVs derived from BV2 cells were increased threefold with HG. Interestingly, in these treatment conditions, when microglia was pretreated 1 h with CsA, CypC content in EVs was significantly reduced. On the other hand, when CypD levels were analyzed, HG treatment did not produce any effect (Fig. 7d). Under inflammation (LPS), the content of CypA, B and C inside EVs was lower than under HG conditions, while CypD levels were significantly increased. In these conditions, CsA pretreatment has no effect on Cyps levels. Nevertheless, CsA control increased four- and two-fold CypB and CypD content inside EVs derived from BV2 cells, respectively.

**Fig. 8:**
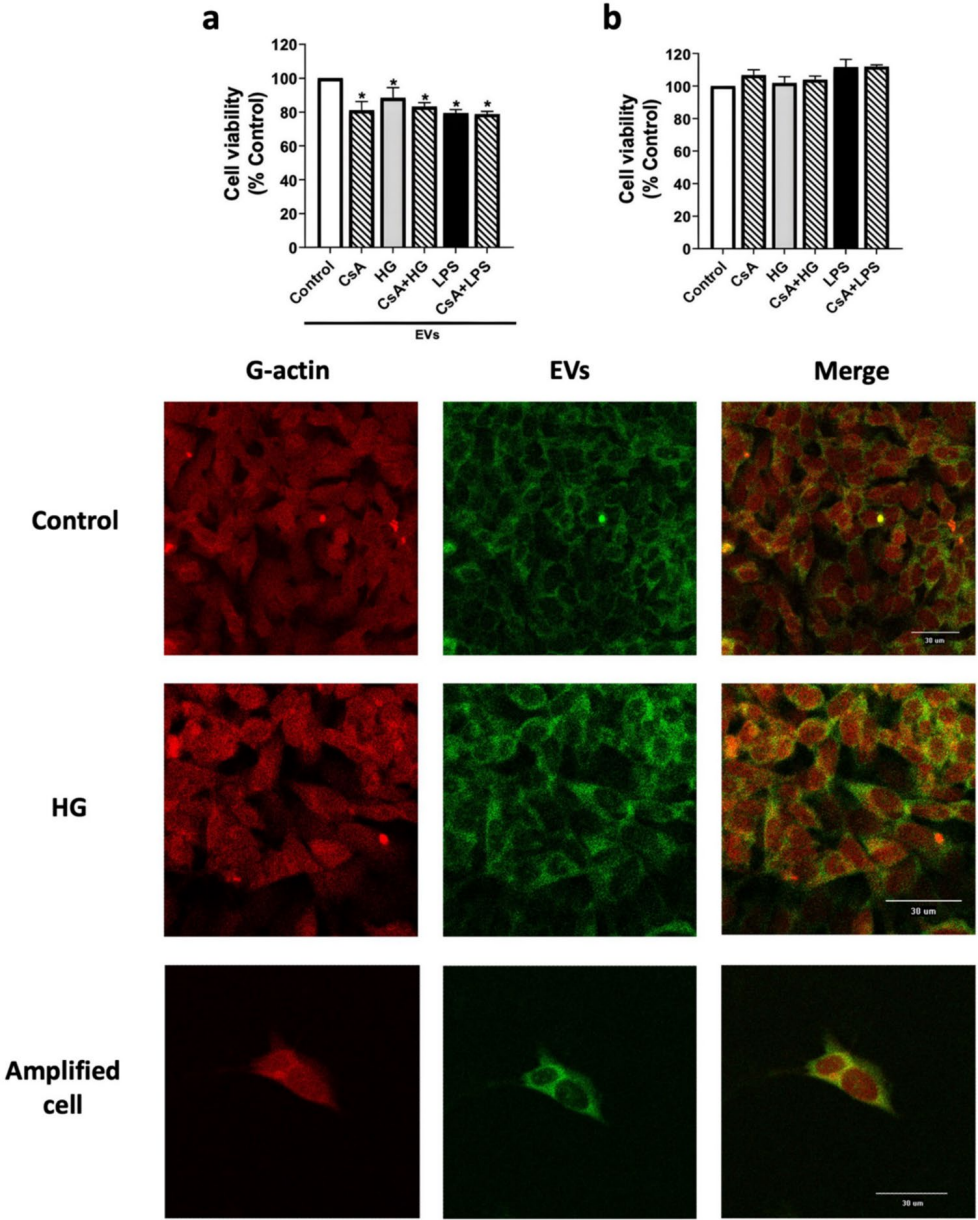
Effect of EVs derived from treated BV2 microglial cells on differentiated SH-SY5Y viability. **a** SH-SY5Y differentiated cells (neurons) were incubated for 24 h with isolated EVs released by microglia cells treated with 25 mM glucose (HG), 1 μM CsA or 500 ng/mL LPS for 24 h. These treatments were also present in neuronal culture media. **b** Effect of treatments in SH-SY5Y differentiated cells (neurons) without EVs. Cell viability was determined by MTT assay. Data are mean ± SEM of three independent replicates. Data are expressed as percentage of control cells. Statistical differences determined by One way ANOVA test and Tuckey’s post hoc test. *p < 0.05 compared to control cells. **c** Confocal images of SH-SY5Y differentiated cells incubated for 24 h with SYTO RNASelect Green-EVs (green) obtained under HG conditions. Texas Red DNase I was used to stain G-actin (red)

Finally, the effect of EVs containing Cyps was analyzed in neurons. In that way, the viability of differentiated and non-differentiated SH-SY5Y neuroblastoma cells was determined after 24 h of treatment with EVs released by BV2 cells under HG conditions. Results show that undifferentiated neuroblastoma cells were unaffected by EVs derived from treated microglia (Supplementary Fig. S4). In contrast, when neuroblastoma was differentiated to neurons and treated with HG-derived EVs, a significant reduction, 20% (*p* < 0.05) in cell viability was observed, the same as under inflammatory conditions (Fig. 8a). Surprisingly EVs released after CsA treatment also decreased cell viability. Neither HG condition, CsA or LPS affected the cell viability of SH-SY5Y differentiated cultured without BV2-derived EVs (Fig. 8b). The uptake of the vesicles by neuronal cells was analyzed by confocal microscopy. BV2-EVs were stained with BODIPY TR Ceramide or SYTO RNASelect Green dyes and added to differentiated SH-SY5Y cells. BODIPY TR Ceramide-EVs uptake was checked after 3 h (Supplementary Fig. S5) and SYTO RNASelect Green-EVs uptake after 24 h (Fig. 8c). After 24 h exposition SYTO RNASelect Green is detected in neurons, either in control or HG condition. Therefore, EVs derived from microglia cells are uptaken by neuronal cells*.* These results suggest that the Cyps contained in EVs released by BV2 cells under HG conditions may have some role in intercellular communication.

In summary, the intracellular expression of Cyps and the levels of the CD147 surface receptor are increased in BV2 microglial cells under hyperglycemia or inflammatory conditions. In addition, an important release of Cyps by EVs is observed. These EVs decreased neurons viability suggesting that this effect is Cyps-mediated.

## Discussion

Neuroinflammation is a response of the CNS to different pathological insults, such as a prolonged state of HG, primary observed on diabetes. The persistence of this inflammatory response contributes to microglial activation, which has important functions during the immune surveillance and plays a pivotal role in the coordination of brain function [53]. During this response, many intracellular pathways are activated and trigger the release of proinflammatory cytokines, chemokines, small molecule messengers, and several proteins including Cyps [40]. As Cyps are related to inflammation, in the present work, the levels of CypA, CypB, CypC and CypD inside microglial cells, in the cell supernatant and their content in EVs under HG conditions are described. As far as we know, this is the first report of EVs release by BV2 microglial cells under HG and of the presence of Cyps inside these cells EVs.

The release of ROS, NO and IL- 6 under HG conditions were reduced in the presence of CsA, which targets Cyps, suggesting an involvement of these proteins in microglial activation. This implication was confirmed when Cyps expression was analyzed, as intracellular levels of CypA, CypB, CypC and CypD were increased in HG-treated microglial cells. These results were also obtained under LPS-associated inflammation, as reported before for CypA [10]. The increased CypA expression observed in HG-activated BV2 cells was also detected in pancreatic β-cells and macrophages. In these cells, CypA inhibition reduced inflammation and oxidative stress, since CypA induces proinflammatory signals, and it is a direct chemoattractant for inflammatory cells [54–57]. CypB enhanced expression under these conditions was described in T lymphocytes from coronary artery disease patients, which had HG levels associated [22, 27]. This Cyp is the only one related to metabolic syndrome, which implies impaired fasting blood glucose or insulin resistance [22]. On the other hand, the increase in intracellular CypC levels in microglia were also detected after ischemia in the brain, a process in which glucose uptake is augmented [58, 59]. Interestingly, CypC was detected at 25 kDa, above its molecular weight (23 kDa), indicating the presence of glycosylated forms of CypC [60]. In the case of CypD, under HG conditions the intracellular expression is enhanced in microglia, as reported on diabetic animals in a model of Alzheimer’s disease transgenic mice [61, 62]. In fact, CypD plays a key role in ischemia–reperfusion injury, atherosclerosis, and diabetes [63–65], and regulates the pore to face a high-fat diet treatment causing mitochondrial dysfunction and insulin resistance [66, 67]. Therefore, an increase in the amount of glucose in the blood could be related to the expression of the mentioned Cyps in microglia.

Given the intracellular levels of Cyps in HG conditions, their extracellular release was also analyzed. HG treatment induces the secretion of both CypA and CypC to the cell medium, while CypB and CypD are undetected. Elevated levels of CypA were found in the serum of patients suffering from type 2 diabetes and in diabetic nephropathy [20, 68, 69]. In the case of CypC, it was described in the serum from coronary artery disease patients in acute phase [43]. Furthermore, in these patients the increased CypC levels were associated with higher HG serum levels [23]. The absence of CypB in the extracellular media is at odds with previous works that reported high CypB serum levels in metabolic syndrome subjects [22]. In concordance with this, another study showed high CypB serum levels in the follicular phase of the menstrual cycle, with glucose concentration increased compared to the other phases of the cycle [24]. CypB was also detected in serum of patients with coronary artery disease, and it was enhanced in those with HG concentration [23]. Therefore, it seems that CypB released to the serum is not related to glia cells. Regarding CypD, there is no data available of its extracellular role, but high CypD levels on cerebrospinal fluid (CSF) and plasma from patients in different inflammatory conditions were described [24, 65, 70].

In view of the lack of CypB and CypD in the cell medium from BV2 cells under HG treatment, Cyps release through a vesicular-dependent mechanism was also analyzed since these cells secrete EVs and CypA and CypD were previously detected inside plasma derived EVs [34–36, 40, 71]. In the present work, Cyps levels are increased in EVs from microglial cells under HG treatment while LPS only increases CypD levels. Differences in EVs content when cells were treated with HG or LPS could be related to the activation of different intracellular pathways by these compounds [5]. Phosphorylation of Extracellular-Signal-Regulated Kinase (ERK) in BV2 cells after LPS remains in basal levels after 24 h treatment [72]. In contrast, 25 mM glucose incubation for 24 h enhanced the activation of this route [73]. ERK is activated when CypA and CypB are overexpressed in cells [55]. Therefore, this kinase activation could be involved in EVs content since Cyps levels are decreased after LPS stimulation while in HG conditions are increased. However, further research is needed to understand which intracellular mechanisms are engaged. As mentioned before, CypA and CypD were previously detected inside EVs derived from patients with different inflammatory diseases [34, 36, 37, 71]. The presence of CypA in EVs under HG conditions could be an alternative secretion route, as it plays an important extracellular role in inflammation [55]. CypD content in EVs could be related with the presence of mitochondria content inside vesicles to sustain mitochondrial homeostasis [74, 75]. Besides, under HG there is an increase on vesicles formation [76]. As far as we know, this is the first description of CypB and CypC release through a vesicular-dependent mechanism under HG conditions, although the release of these Cyps through the endocytic pathway was previously pointed [60]. However, CypB was detected in plasma from metabolic syndrome subjects [22], so it could be that under HG conditions it is also secreted by another way. Regarding CypC, this protein has a key role in cardiovascular pathologies, which are related to HG [23, 43]. CypC was again detected above its molecular weight, suggesting that microglia releases the glycosylated CypC form through EVs [60]. CsA pretreatment only reduces the release of CypA and CypC through EVs when cells are under HG conditions while the content of CypB and CypD inside EVs is increased. This suggests that Cyps inhibition by CsA may be promoting the secretion of Cyps. CypB secretion by CsA through the constitutive pathway was described in keratinocytes [77]. This is the pathway where EVs are mainly originated. Moreover, CypB is an ER-resident immunophilin, which is the organelle where vesicular traffic begins [60]. Therefore, the increased levels of CypB in EVs after CsA treatment could be due to the secretion through this pathway. Regarding CypD, there is a relationship between mitochondria (CypD reservoir) and lysosomes, part of the constitutive secretory pathway, in neurodegenerative disorders so CypD could be released in the same way as CypB [78]. Nevetheless, these results are unexpected, and we have no explanation as to why only CypB and D show this pattern. In fact, it is also surprising that CsA decreases the levels of all Cyps in HG conditions while no effect is observed when cells are LPS treated. This point again to the activation of different intracellular pathways. When the effect of EVs released by microglial cells and HG conditions was evaluated in neurons, the viability of these cells was significantly compromised in all the treatments checked since microglial vesicles are uptaked by neurons. This effect is probably mediated by Cyps EVs-content, in HG treated cells CypA, CypB and CypC-mediated, while CypD-mediated in LPS treatment, since EVs derived from microglia can be uptaken by neurons. Moreover, the concentration of EVs was increased after 25 mM glucose or LPS treatment, therefore under inflammatory conditions glia cells release a high amount of Cyps-containing EVs, which could contribute to reduce the neuronal viability. The toxic effect of EVs released after CsA treatment observed in neurons is in line with the important neurotoxic effect of this compound widely described in literature [79]. From our results some relationship with CypB and CypD could be concluded. The effect in differentiated SH-SY5Y cells suggests that Cyps released from microglia reduce neuronal viability under pathological conditions such as hyperglycemia or inflammation and points to some role of these proteins in cellular communication.

As previously described, CD147 is the receptor for extracellular CypA and CypB. When high levels of Cyps are detected, the CD147 receptor is externalized to the cell surface to induce the cell response associated to Cyps [80]. The extracellular receptor of CypC remains unknown, although it was described a binding site in CD147 for CypC [26, 81]. In consequence, given the increase of extracellular Cyps levels under HG, the presence of CD147 in the surface of microglia was analyzed. The expression of CD147 surface membrane receptor is upregulated by HG treatment, the same as under other inflammatory conditions [17]. In microglial cells, it was observed that LPS treatment increases the expression of CD147 [10], as reported in this work. Interestingly CD147 receptor expression matches with intracellular increase of Cyps expression after HG or LPS treatment and decreases when CsA is present, pointing again to intracellular Cyps expression as the main factor for CD147 externalization. There is no data available of hyperglycemia effect on CD147 receptor levels in microglia. However, a recent study described an increase on CD147 receptor secretion in monocytes and endothelial cells after HG treatment [47]. In fact, glucose uptake, which is controlled by glucose transporter 1 (GLUT1) is increased by CD147 receptor [82]. These results, together with the presence of Cyps in the extracellular medium, suggest that CD147 and Cyps participate in the inflammatory response associated to high levels of glucose, primary observed in many cardiovascular and neurodegenerative diseases [2, 3].

The neuroinflammatory response by microglia underlying HG leads to systemic insulin resistance and type 2 diabetes mellitus [83]. Diabetes mellitus in one of the largest global public health concerns and Cyps could be early signals in HG-associated pathologies such as Alzheimer's disease or cardiovascular diseases.

## Conclusion

Thus, Cyps and its receptor play a complex role in the inflammatory response underlying HG. This work is the first study that shows a direct connection between Cyps, EVs and microglia. Results indicate that hyperglycemia induces the intracellular expression of CypA, CypB, CypC and CypD and their release through a vesicular-dependent mechanism, which seems to be different depending on the inflammatory stimuli. Moreover, EVs from HG-treated microglia affect the viability of neuronal cells, suggesting an important role of Cyps in brain function in pathological conditions. The present results in microglial cells strengthen the role of Cyps under hyperglycemia conditions, pointing to Cyps as potential biomarkers of pathologies associated with high levels of glucose such as cardiovascular diseases, cancer, covid- 19 and diabetes [84–89]. In all these processes, Cyps could be promising targets. Therefore, to disclose the relationship between Cyps and hyperglycemia could greatly contribute to the diagnosis, prognosis and treatment of many diseases with great impact in our society.

## Supplementary Information

Below is the link to the electronic supplementary material.

Supplementary file1 (PDF 3342 KB)

Supplementary file2 (PDF 11238 KB)

## Data Availability

No datasets were generated or analysed during the current study.

## Abbreviations

CNS: Central nervous system
CsA: Cyclosporin A
CSF: Cerebrospinal fluid
Cyps: Cyclophilins
CypA: Cyclophilin A
CypB: Cyclophilin B
CypC: Cyclophilin C
CypD: Cyclophilin D
ER: Endoplasmic reticulum
ERK: Extracellular-signal-regulated kinase
EVs: Extracellular vesicles
FBS: Fetal bovine serum
GLUT1: Glucose transporter 1
HG: High glucose
ICLC: Interlab cell line collection
LPS: Lipopolysaccharide
MAPK: Mitogen-activated protein kinase
MTT: 3-(4,5-Dimethylthiazol- 2-yl)- 2,5-diphenyl tetrazolium bromide
mPTP: Mitochondrial transition permeability pore
NO: Nitric oxide
NTA: Nanoparticle traficking analysis
PBS: Phosphate buffered saline
PPIase: Peptidyl-Prolyl *cis–trans* isomerase
PVDF: Polyvinylidene difluoride
ROS: Reactive oxygen species
SDS: Sodium dodecyl sulfate
TEM: Transmission electronic microscopy
