# Supplementary material for: Cyclophilin A and C are the Main Components of Extracellular Vesicles in Response to Hyperglycemia in BV2 Microglial Cells
Source: Mol Neurobiol. 2025 Apr 8;62(8):10349–66. doi: 10.1007/s12035-025-04921-6 (PMC12289835; doi:10.1007/s12035-025-04921-6)
Supplement: Supplementary file 1 — Supplementary file1 (PDF 3342 KB) [file 12035_2025_4921_MOESM1_ESM.pdf]

# Supporting Information

**Cyclophilin A and C are the main components of extracellular vesicles in response to hyperglycemia in BV2 microglial cells**

Noelia Castedo[1], Amparo Alfonso[1], Rebeca Alvariño[2*], Mercedes R. Vieytes[2], Luis M. Botana[1*]

[1]Departamento de Farmacología, Facultad de Veterinaria, IDIS, Universidad de Santiago de Compostela, Lugo 27002, España

[2]Departamento de Fisiología, Facultad de Veterinaria, IDIS, Universidad de Santiago de Compostela, Lugo 27002, España

*Correspondence: rebeca.alvarino@usc.es; luis.botana@usc.es

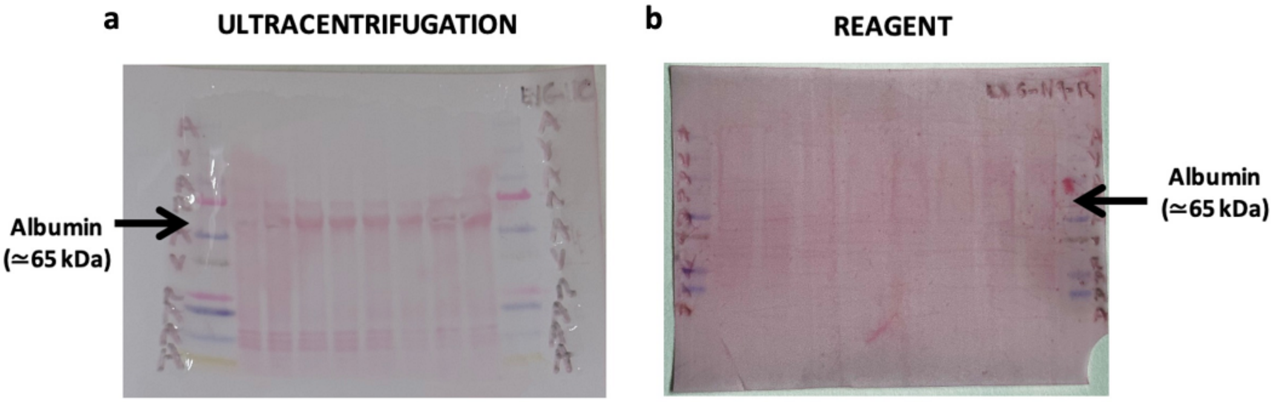


**Fig.S1.** Ponceau Tinction. Membranes with ultracentrifugation (a) and reagent (b) samples were dyed with the Ponceau Tinction to visualize Albumin (65 kDa).

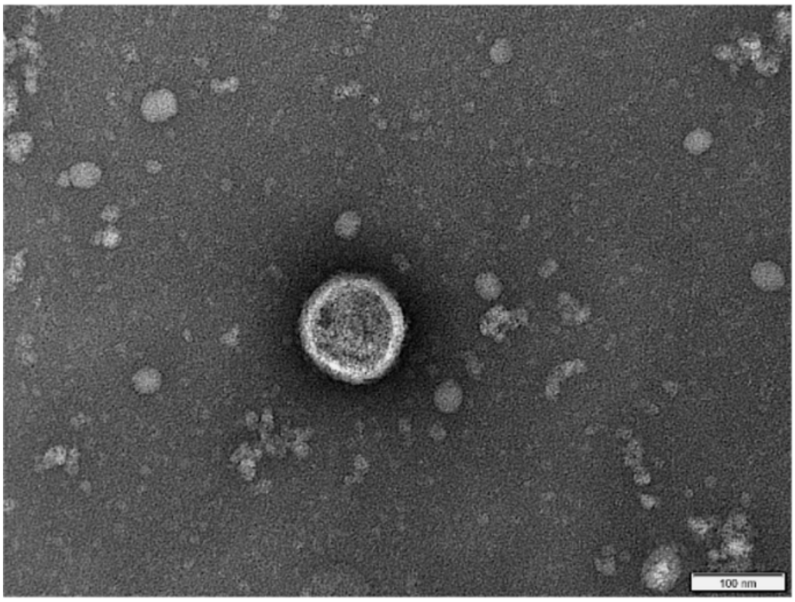


**Fig.S2.** Characterization of EVs derived from BV2 microglial cells. EVs presence determined through visualization by TEM. A scale of 100 nm was used.

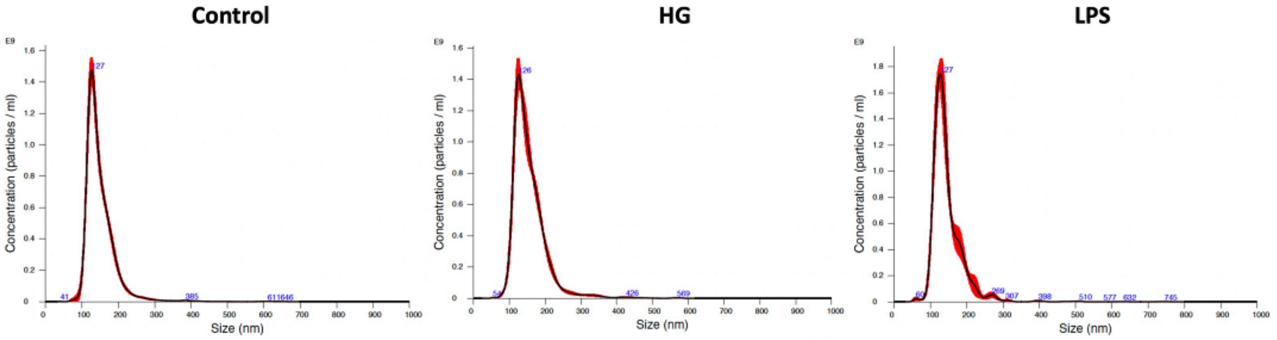


| | Control | HG | LPS |
|---|---|---|---|
| **Size (nm)** | 150.3 ± 0.9 | 155.5 ± 0.7 | 145.6 ± 0.5 |
| **Concentration (particles/mL)** | 8.30e+10 ± 2.18e+09 | 1.03e+11 ± 1.35 e+09 | 1.03e+11 ± 1.40e+09 |

**Fig.S3.** Characterization of EVs derived from BV2 microglial cells. Nanosight analysis of particle size distribution and concentration from control EVs, HG and LPS treated EVs.

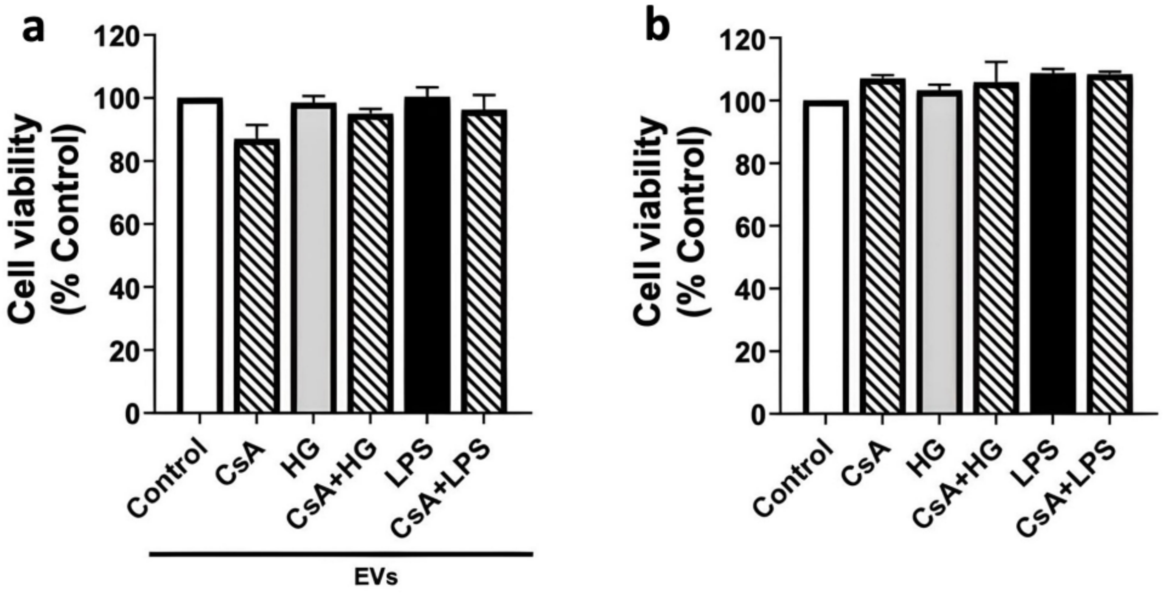


**Fig.S4.** Effect of EVs derived from treated BV2 microglial cells on SH-SY5Y cell viability. a) SH-SY5Y neuroblastoma cells (non-differentiated) incubated for 24 hours with isolated EVs released by microglia cells treated with 25 mM glucose (HG), 1 µM CsA or 500 ng/mL LPS for 24 hours. b) SH-SY5Y neuroblastoma cells (non-differentiated) incubated with all the treatments without EVs. Cell viability was determined by MTT assay. Data are mean ± SEM of three independent replicates. Data are expressed as percentage of control cells. Statistical differences determined by One way ANOVA test and Tuckey's post hoc test. *p<0.05 compared to control cells

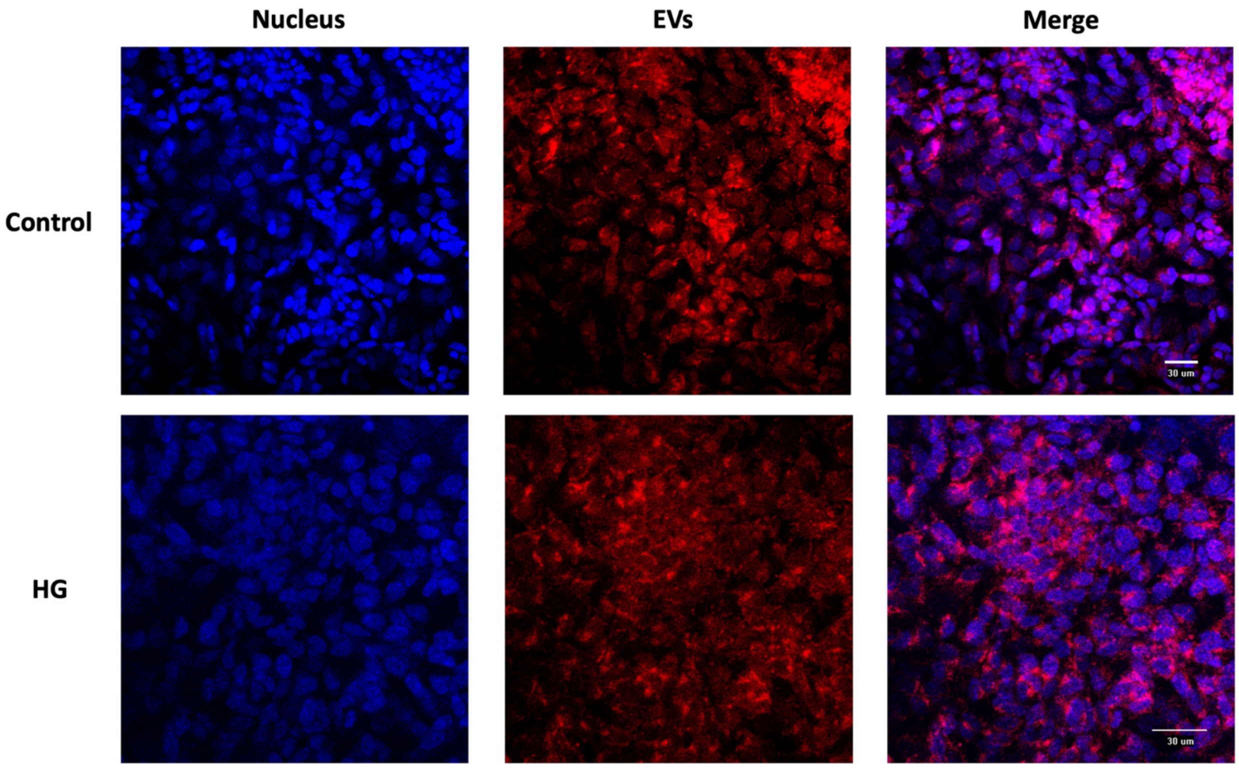


**Fig.S5.** Confocal images of SH-SY5Y differentiated cells incubated for 3 hours with BODIPY TR Ceramide-EVs (red) obtained under HG conditions. Hoechst was used to stain nucleus (blue).
