# Supplementary material for: Cyclophilin A and C are the Main Components of Extracellular Vesicles in Response to Hyperglycemia in BV2 Microglial Cells
Source: Mol Neurobiol. 2025 Apr 8;62(8):10349–66. doi: 10.1007/s12035-025-04921-6 (PMC12289835; doi:10.1007/s12035-025-04921-6)

Supplementary Information

Original western blot images

Figure 3a

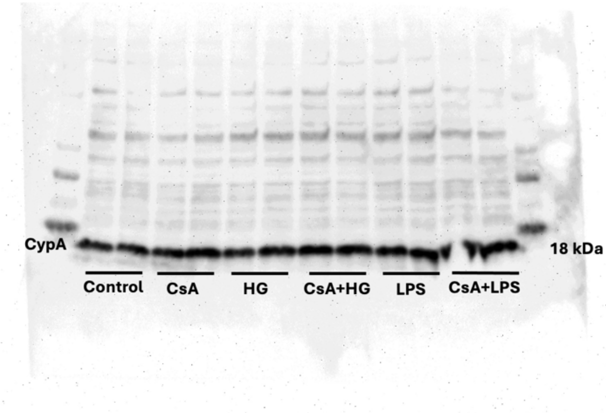

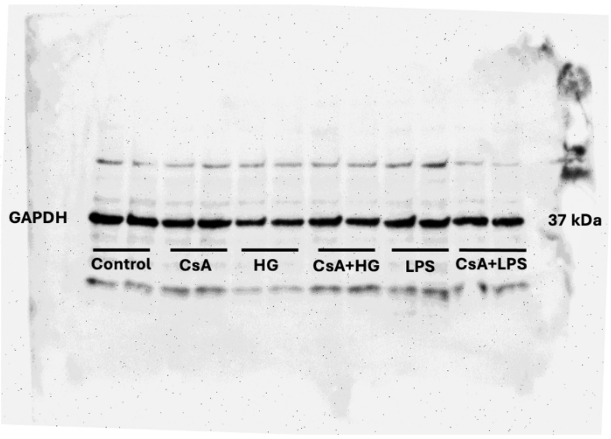

Figure 3b

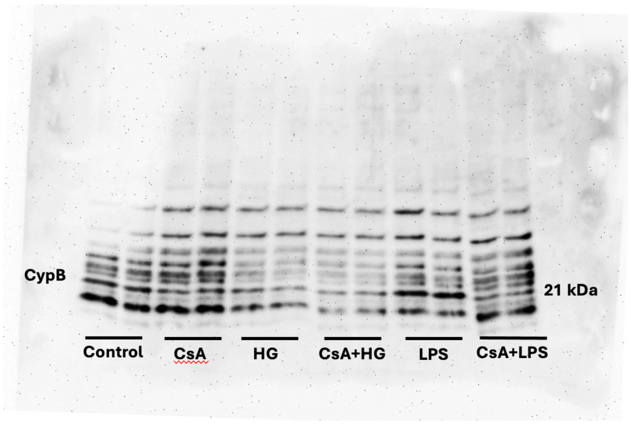

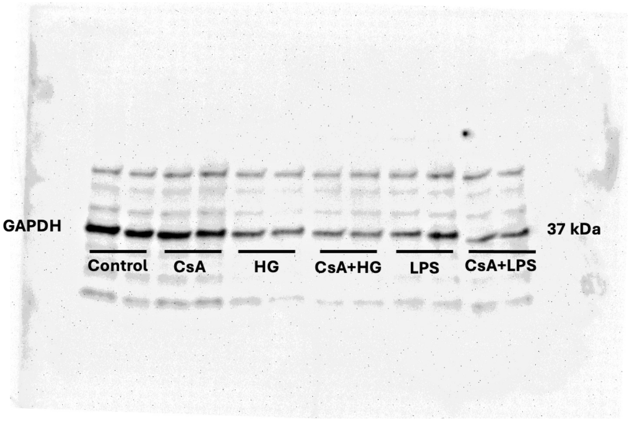

Figure 3c

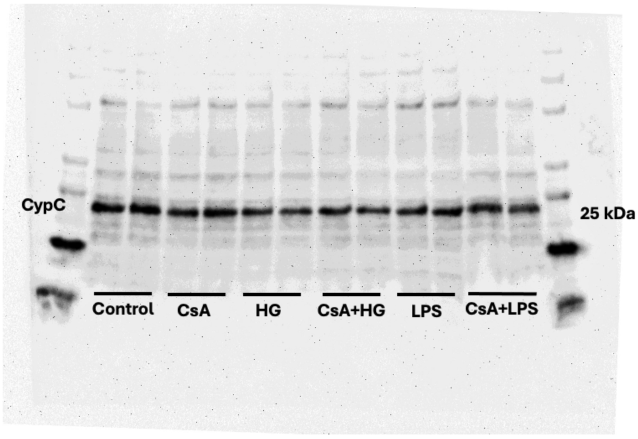

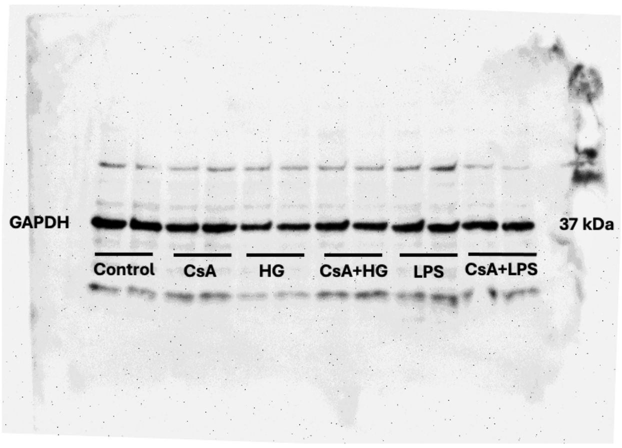

Figure 3d

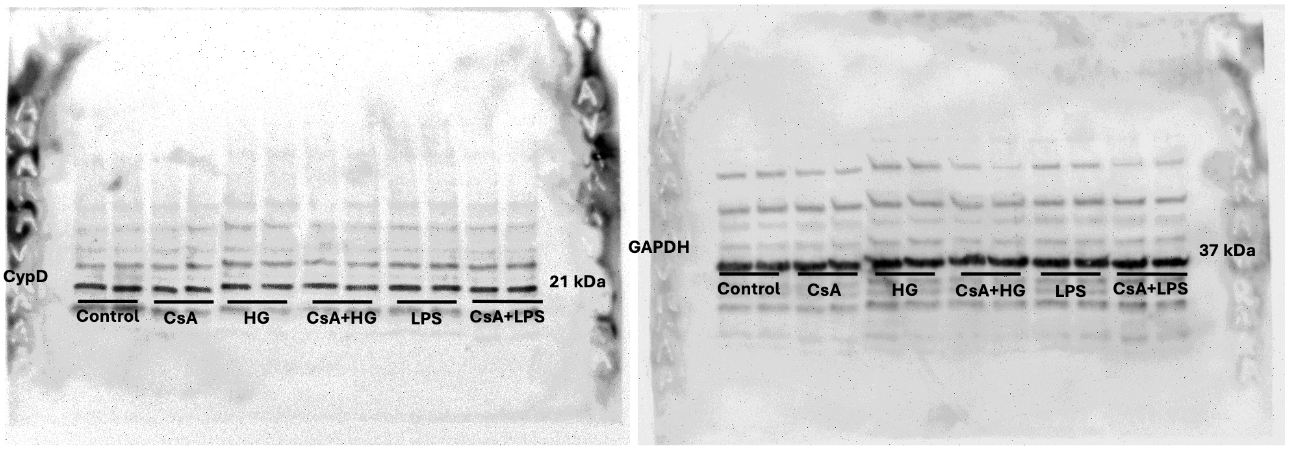

Figure 5

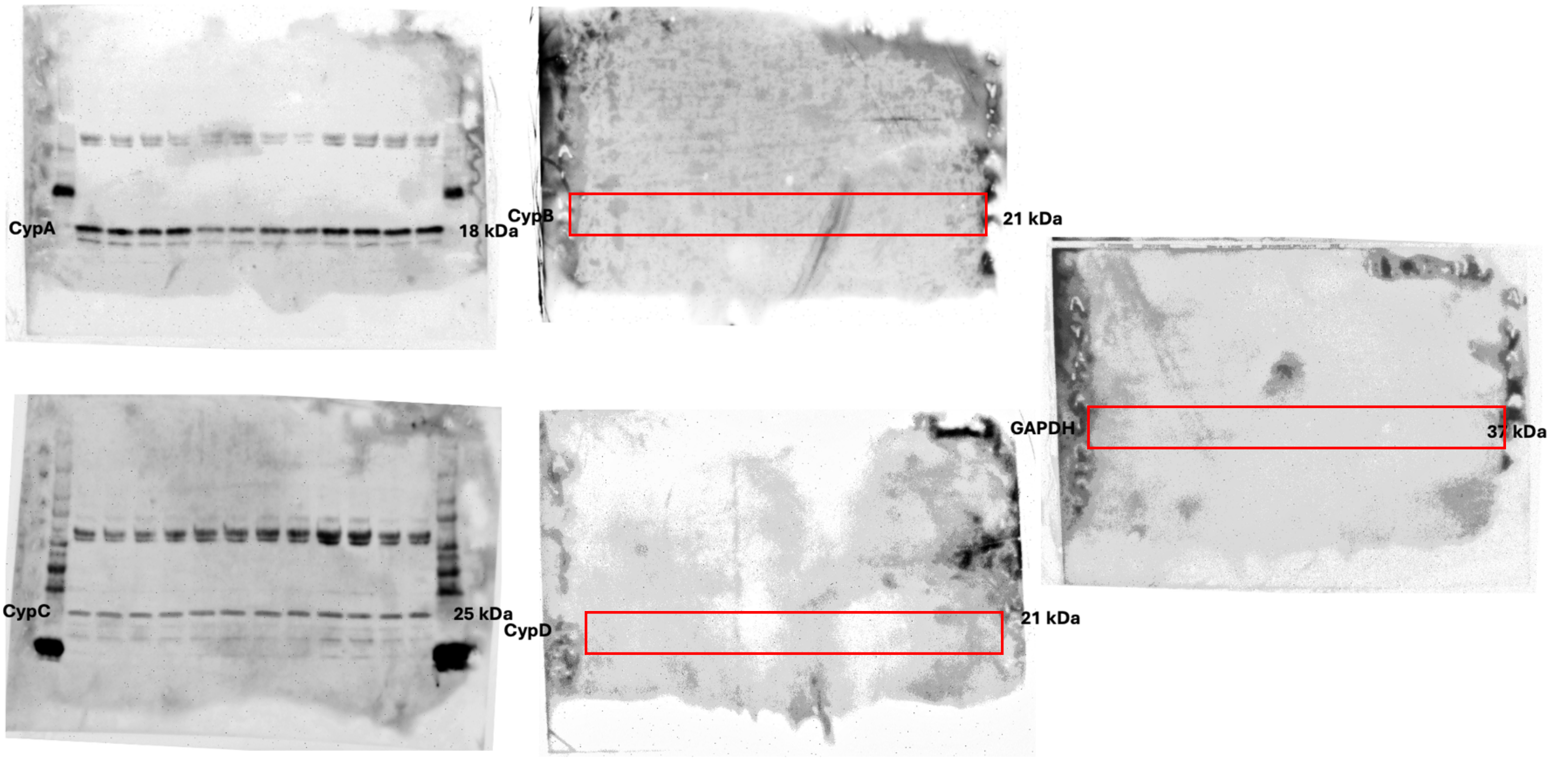

Figure 6

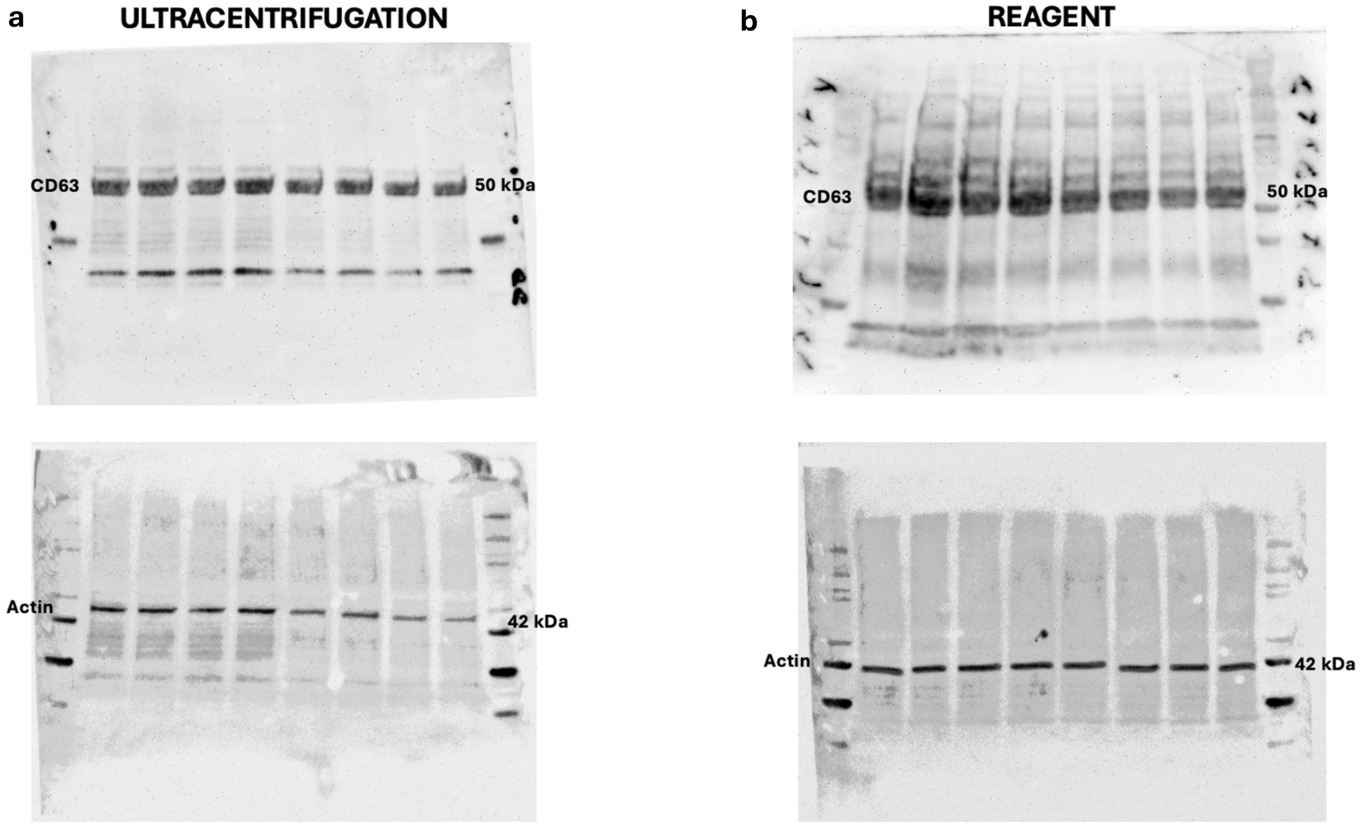

Figure 7a

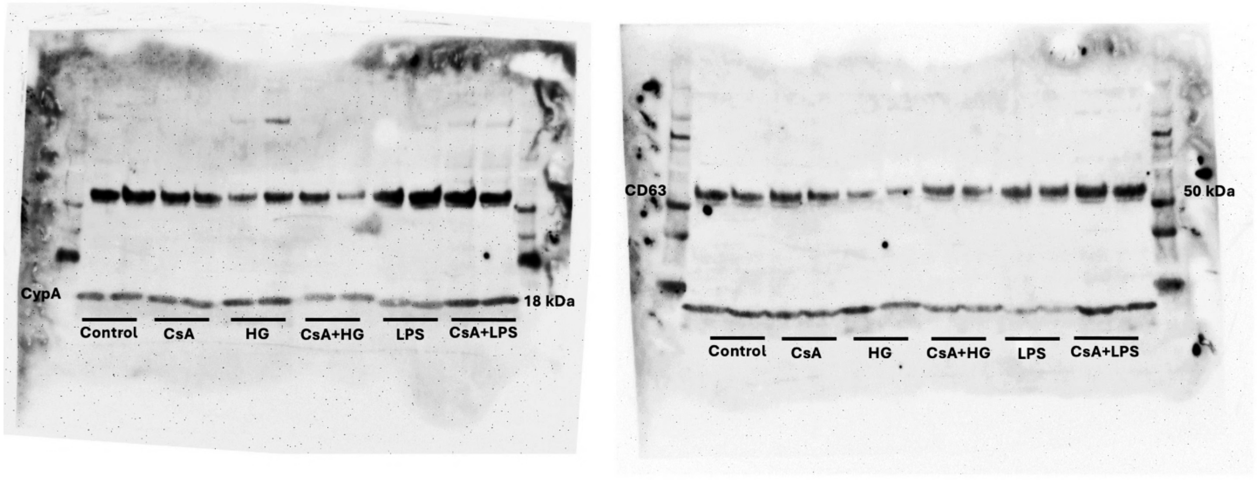

Figure 7b

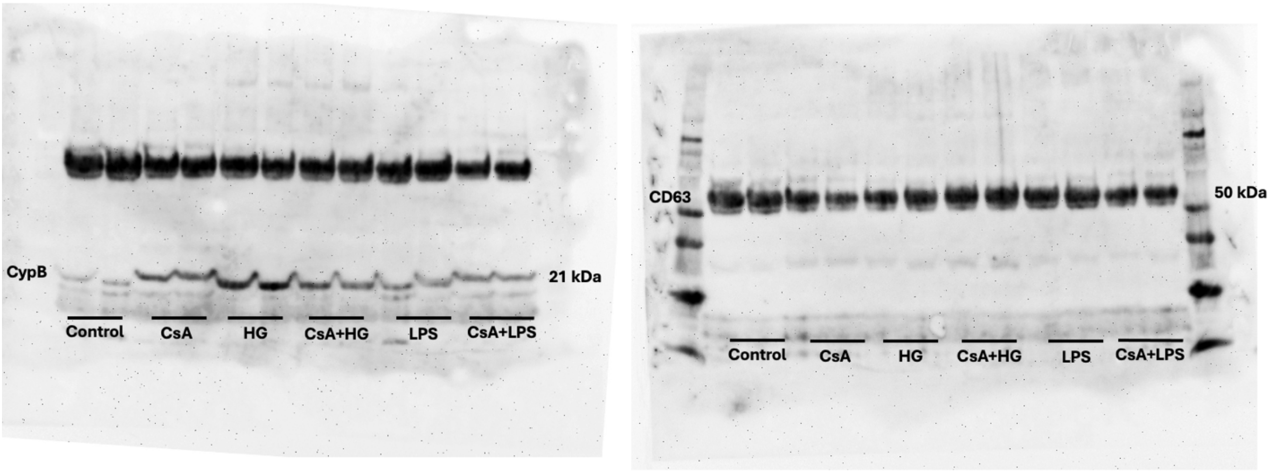

Figure 7c

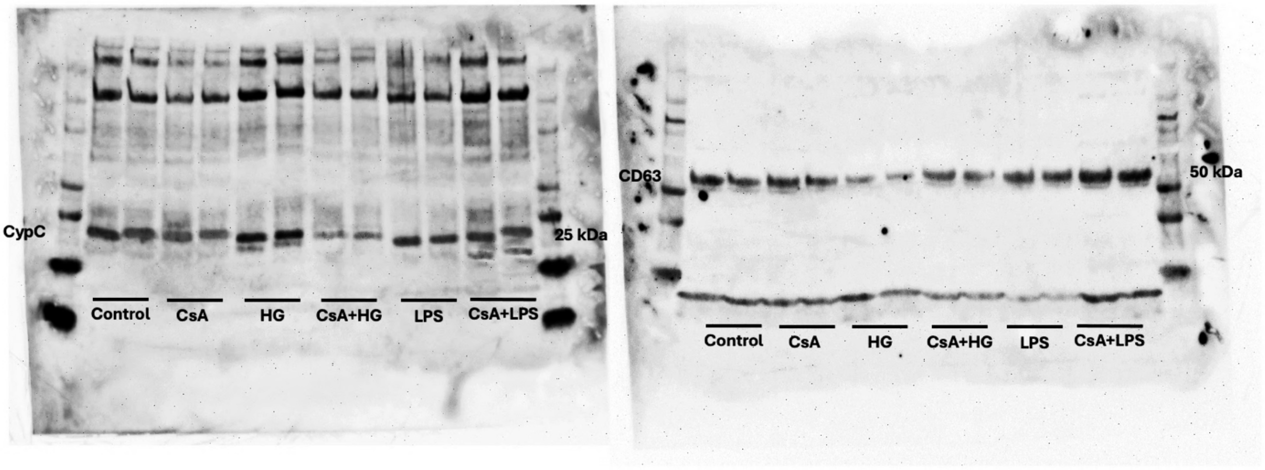

Figure 7d

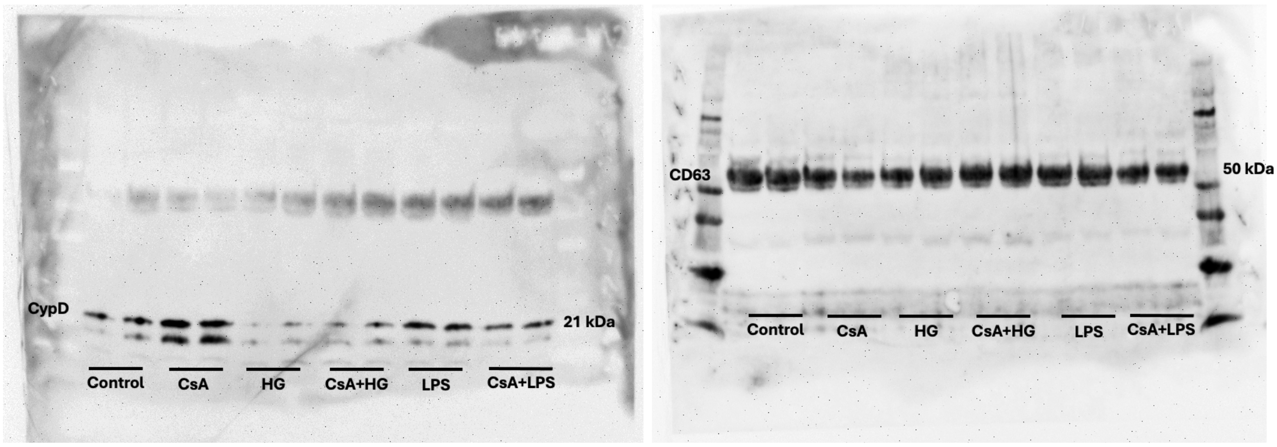

Supplement: Supplementary file 2 — Supplementary file2 (PDF 11238 KB) [file 12035_2025_4921_MOESM2_ESM.pdf]
